# Cross-border contextualisation of family medicine curriculum: an examination of the process

**DOI:** 10.5116/ijme.64e3.740e

**Published:** 2023-08-31

**Authors:** Brian McEllistrem, Molly Owens, David L Whitford

**Affiliations:** 1 Irish College of General Practitioners, 4/5 Lincoln Place, Dublin 2, Ireland; 2 RCSI & UCD Malaysia Campus, 4 Jalan Sepoy Lines, 10450 Georgetown, Penang, Malaysia

**Keywords:** Curriculum, Delphi technique, general practice, family medicine, cross-border

## Abstract

**Objectives:**

This study explores a method of transferring a post graduate medical education curriculum internationally and contextualising it to the local environment. This paper also explores the experiences of those local medical educationalists involved in the process.

**Methods:**

Several methods were implemented. Firstly, a modified Delphi process for the contextualisation of learning outcomes was implemented with a purposefully sampled expert group of Malaysian Family Medicine Specialists. Secondly a small group review for supporting materials was undertaken. Finally, qualitative data in relation to the family medicine specialists' experiences of the processes was collected via online questionnaire and analysed via template analysis. Descriptive statistics were used.

**Results:**

Learning outcomes were reviewed over three rounds; 95.9% (1691/1763) of the learning outcomes were accepted without modification, with the remainder requiring additions, modifications, or deletions. Supporting materials were extensively altered by the expert group. Template analysis showed that Family Medicine Specialists related positively to their involvement in the process, commenting on the amount of similarity in the medical curriculum whilst recognising differences in disease profiles and cultural approaches.

**Conclusions:**

Learning outcomes and associated material were transferable between "home" and "host" institution. Where differences were discovered this novel approach places "host" practitioners' experiences and knowledge central to the adaptation process, thereby rendering a fit for purpose curriculum. Host satisfaction with the outcome of the processes, as well as ancillary benefits were clearly identified.

## Introduction

The exponential growth in cross-border partnerships in medical education^1^ has seen both negative and positive impacts. This spectrum of positions has also been called hyperglobalist (positive) and sceptical (negative).^2^ Negative impacts range from the movement of information, including curriculum development, being seen as "quite unidirectional, going from Europe and North America" ^3^ to more pejorative indictments of the relationship between the home and host institutions - "We have a master-slave relationship" .^4^ Subtler organisational elements, such as ownership of the institutions lying with the home country, have led authors to drawing parallels with colonial exploitation.^5^ Notwithstanding the above difficulties, positive developments have been seen. Pathways towards equal partnerships between home and host institutions have been described via faculty roles being shared and/or transitioned to local educators.^3^^,^^6^ Additionally, educators at host institutions have reported a feeling of connectivity with the home institution and that the positions they held were beneficial to their careers.^7^ A key theoretical framework of any cross-border medical education initiative is the "contextualisation of the curriculum" ^6^ and acknowledging "the tension between ensuring both equivalence across sites and contextualisation to suit the local context".^8^

RCSI & UCD Malaysia Campus (RUMC) was invited in 2017 to establish a new specialist training programme in Malaysia for Family Medicine Specialists (FMS), also known as general practitioners, in collaboration with the Irish College of General Practitioners (ICGP) and a third-party medical education company. The MInTFM programme is a four-year programme, modelled on the ICGP programme and designed to utilise the contextualised curriculum and assessments. There was a clear aim from the outset to avoid the "copy-pasting" ^9^ deficiencies seen in previous cross border partnerships, whilst incorporating the beneficial approaches described in the literature and retaining the refinements and established mechanisms of the ICGP curriculum.

The aim of this study was to examine a novel process of cross-border curriculum contextualisation. The objectives were to examine a modified Delphi process for contextualising learning outcomes, examine the process of contextualising the support materials, and finally explore the experiences of the expert group, also "host" medical educators, of the process.

## Methods

### Study Design

Three separate methods were applied during the contextualisation process: a modified Delphi process, an FMS co-authorship model, and a subsequent exploration of the FMS experiences of the process (completed via qualitative feedback from FMS participants). The processes commenced in April 2019 and completed in February 2021.

The ICGP Curriculum for GP training in Ireland^10^ consists of 34 chapters, 29 of which relate to clinical topics and 5 to non-clinical topics. Each chapter consists of an introduction, a case vignette, and associated reflective questions, learning outcomes, resources, and references. The learning outcomes (LO) and reflective questions are organised via the 6 core competencies and 3 core skills of the World Organization of National Colleges, Academies and Academic Associations of General Practitioners/Family Physicians (WONCA) framework within each chapter.^11^

### Participants

Purposeful sampling was implemented to form the expert group which consisted of 18 experienced FMS based in Malaysia. This study was granted a letter of exemption from the Joint Penang Independent Ethics Committee, RCSI & UCD Malaysia Campus.

#### Modified Delphi process for Learning Outcomes

The classic Delphi process is described as "a method used to obtain the most reliable consensus of opinion of a group of experts by a series of intensive questionnaires interspersed with controlled feedback".^12^ The process implemented for the MInTFM curriculum was a modified Delphi process, as the existing 1,763 LO of the ICGP curriculum formed the initial dataset.

A three round Delphi process was agreed to ensure timely completion and was supported by two early pilot rounds which exhibited high levels of concordance. The LO were sent out chapter by chapter to the expert group, via an online survey tool.^13^ The expert group had two weeks to complete each round, with each chapter requiring up to three rounds. Rounds ran concurrently, with the first round of one chapter often being reviewed the same weeks as second or third rounds of other chapters.

Regular updates were provided to the Delphi group. This allowed for both transparency of progress for the first two stages and also "nudges" to complete allocated work for those underperforming in relation to their peers.^14^

#### Round One

All LO from the ICGP curriculum were appraised. Two options per LO were presented, either to accept the LO as existing or alter/delete it. Each LO also had a free text entry "Alternative LO suggestion" to allow modifications to be entered by the expert group members.

Those LO reaching greater than 80% acceptance, within the expert group, were incorporated into the MInTFM Curriculum in their respective chapter and WONCA domain. Those LO with less than 80% acceptance were brought forward to round two, with suggested modifications from their respective free texts if available. In addition, the expert group were invited to add new LO.

#### Round Two

The research lead, informed by the round one results of new LO suggestions and modifications to existing LO with less than 80% acceptance, generated a new list of LO for expert group review. Each learning outcome had 3 options in round two (and free text for modification suggestion); accept the new version of the LO, further alter it, or delete it. The option to delete was made available at this point so as to allow early feedback to be incorporated before LO were potentially removed. LO at or above 80% for acceptance/deletion were appropriately actioned. Others not meeting these two criteria were brought forward to round three.

#### Round Three

Round three only differed from round two in that those LO which did not reach 80% acceptance/deletion were to be forwarded to the MInTFM Curriculum Sub-Committee. This eventuality did not occur.

#### Contextualising the supporting material

All material within each chapter that was not a learning outcome was treated as supporting material. This included the introduction, case vignette and reflective questions, resources, and references. These sections were introduced to the 2016 version of the ICGP curriculum in order to assist all in the training community to better use the curriculum, the previous iteration of which had been solely a list of learning outcomes. For this part of the contextualisation process, the 18 FMS, working in pairs, were allocated chapters to review and rewrite, as necessary.

#### Exploring FMS experience of contextualisation

To explore the Malaysian FMS experiences of the contextualisation process, we chose an explorative phenomenological approach.^15^ The whole FMS modified Delphi group was invited to complete the survey. Qualitative feedback was collected via four questions, available in Appendix, hosted as an online survey.^13^ To identify themes and sub-themes across the free text feedback a template analysis method was used, as described by King.^16^ Analysis of all feedback was independently performed by first author and another author and concordance agreed for themes and subthemes. To address reflectivity, the contextualisation concepts and processes were regularly discussed amongst the paper's co-authors.

## Results

The modified Delphi process for learning outcomes resulted in all chapters completing round one, with 24 chapters proceeding to round two and seven to round three. 95.9% (1691/1763) of the learning outcomes were accepted without modification, with the remainder requiring additions, modifications, or deletions. The response rate of the expert group (n=18), over all rounds of the Delphi process, was 78% (14/18) which is acceptable.^17^^,^^18^ Eight of 1763 (0.45%) LO were added. All inserted LO were generated to reflect pathologies or management options not common in Ireland.  Modifications to LO were needed in 3.1% (55/1763) cases. Of these changes, 53% (29/55) were required to reflect the differing disease profile of primary care encountered in Malaysia. The remaining changes reflected non-disease profile issues such as differing cultural considerations and administrative practices.  A situation arose in relation to one LO pertaining to female genital practices. These practices are known as mutilation and circumcision depending on the cultural context. This LO garnered significant and emphatic free text feedback at round one and a low acceptance rate of 50% (9/18). For this LO the project lead was guided by the suggestions of the two incoming Malaysian programme directors regarding the wording of the LO. This was done with iterative rounds of refinement via email correspondence, and it was accepted at round two at 88% (16/18). 0.51% (9/1763) LO were deleted during the process, with the majority, 89% (8/9), being corrections of existing duplications in the curriculum, and the remaining percentage pertaining to a specific health body contract.

Thirty-four chapters had their introductions, case vignettes, reflective questions, resources, and references, re-written where necessary. In total 167 Malaysian specific resources were added and 37 Malaysian specific references. Nine FMS completed the survey exploring their experience of the contextualisation process. Template analysis generated two overarching themes with associated subthemes, as can be seen in Table 1. These two themes are illustrated with supporting FMS excerpts in the following sections.

**Table 1:** Template Analysis Overview

Template Analysis overview
1. Contextualisation process
a. Importance and international collegiality
b. Novel learning experience
c. Comparing and contrasting academic systems
2. Primary Care
a. Universality.
b. Local context differences.
i. Direct pathology related.
ii. Cultural/societal/religious/health system

### Theme 1: Contextualisation process

The first overarching theme explores the process itself from the FMS perspective, with further areas focused on within the three subthemes. In the first subtheme the education imperative of a suitable curriculum was acknowledged - "we find it is important to make sure the contents of the course [are] applicable to the local Malaysia setting", with the process of working with international colleagues also seen as an advantage by "sharing and exchanging of views on certain aspects. Working on it as a team and being guided by the Irish team made it easier". The nature of reviewing 1,763 LO was mentioned as "tedious", however this was counter balanced with the process being described as "systematic and comprehensive". The second subtheme looked at the contextualisation in relation to the personal development of the FMS both as medical educators and physicians. FMS found the process "an eye opener" which was "rewarding & good learning experience on personal level", with the process also carried over to clinical practice development - "[the] WONCA (Europe) curriculum framework in approaching case management for primary care providers is helpful". The third subtheme compared the FMS experiences of the existing postgraduate training pathways to FMS with MInTFM. MInTFM was seen as having "similarities of the curriculum to the Malaysian Masters" and "similar to the local universities family medicine master program", with "minor variations" still existing.

### Theme 2: Primary care

The second theme focused on the speciality of primary care. Firstly, it was acknowledged that overall, significant similarities in primary care exist between the two jurisdictions, with the "majority of the basics … similar and universal to both countries". The dissimilar content can be delineated into two areas: firstly, differing medical pathology presentations and secondly, non-pathology related considerations. In relation to pathology considerations, FMS cited that "several medical conditions may not be prevalent in the Malaysian & vice versa". Secondly, FMS experiences on how to represent religious and similar topics in the curriculum formed part of the contextualisation process - "majority of Malaysian are Muslim, so they have additional pilgrim health on the curriculum".

## Discussion

Our curriculum contextualisation process has found that general practice, in respect of post-graduate curricular learning outcome considerations, is very similar in Ireland and Malaysia, with 95.9% (1691/1763) of learning outcomes being mutually acceptable. However, in a limited number of cases considerable adaptations are required to reflect local context. These adaptations range from issues such as disease prevalence and associated presentations within primary care, to more sensitive areas of cultural and religious beliefs. Similar areas have been seen in the literature with differing opinions held in relation to the acceptability of cultural and/or religious activities.^19^ Within our processes the learning outcome pertaining to female genital practices had the most emphatic responses. This difference in terminology reflects the prevalence of this practice among Muslim communities in Malaysia^20^ and the legislative position in Ireland in relation to the practice – which make it a criminal offence to leave Ireland to carry out such practices.^21^ Yu cited similar dissimilarities in other sensitive areas of culture namely death, as in Western culture there is a belief in the afterlife, whilst in Chinese culture death is life lost forever.^22^ Our process mirrors that of others in similar cross border postgraduate training programs in that there is a ready alignment of the medical science between two jurisdictions but not necessarily a similar alignment of cultural and societal considerations.^23^

The literature describes the considerable range of both cautionary tales and successes which must be considered in order to establish an equitable and productive cross-border educational initiative – with more modern approaches acknowledging this with the new perspective of "transformationalist".^2^ The transformationalist perspective acknowledges both the positive and negative realities of cross border processes. It also promotes combining international and local approaches,^24^ modifying for local context^26^ and retaining local culture as a central importance.^26^ We have described in this paper our approach to addressing these areas, which include negative overtones between the home and host institutions, whilst serendipitously capitalising on positive opportunities. By systematically and deliberately involving the Malaysian FMS in all steps of the modified Delphi process, we ensured a host educator centred approach from planning, through application to completion in order to build an onward trajectory towards delivering training.^27^^,^^29^

Feedback from our FMS group acknowledges our shared understanding of the high level of importance of curriculum contextualisation, which is further reinforced by the high participation rate. This process had an additional benefit of building a sense of collegiality, a recognised positive experience in the cross-border education literature.^3^^,^^6^ The process has also created a shared vision for the new MInTFM programme from a guiding coalition of empowered team members.^29^ These views are mirrored in the FMS experiences of the contextualisation process. We have also hopefully sown the seeds for the transfer of academic responsibilities from home to host institution, by deeply familiarising future FMS trainers and programme directors with the curriculum.

### Limitations

This project has several limitations.  Firstly, a classic Delphi was not implemented, as the initial dataset was provided. However, McKenna^30^ described 7 key characteristics of the Delphi process, 6 of which were followed for this paper. The remaining characteristic "The use of frequency distributions to identify patterns of agreement" was replaced with the project lead, who, whilst reviewing the replies of the rounds, sought associations between the free text (per LO) modification suggestions and new LO suggestions. These associations formed the basis for alternate/new LO in subsequent rounds of the Delphi. A second limitation was that a de novo curriculum was not created, instead an existing curriculum was adapted. This choice stemmed from the premise of the ICGP being invited to deliver primary care specialist training based on its already established training practices which included the curriculum. Finally, the processes and content were completed in the English language, which may have added complexity to the process, despite being widely used amongst doctors in Malaysia.^31^^,^^32^

## Conclusions

This paper describes the similarities and differences discovered during the novel processes and protocols which were implemented to contextualise the Irish curriculum for GP training for the newly launched FMS training scheme in Malaysia – MInTFM, and it also explores the FMS experiences of same. Overall, the vast majority of LO at 95.9% (1691/1763) did not need alteration, indicating a high degree of similarity in the practice of family medicine between the two countries. However, for the remaining LO appropriate changes/additions/removals were made and were guided by host FMS. This contextualisation is one approach which prioritises the input and expertise of practitioners in the host country with academics from the home institution providing a framework and guidance when appropriate. The changes to the ICGP curriculum should ensure that the MInTFM curriculum is fit for purpose and unique to Malaysia, and therefore able to train graduates for independent practice as FMS in Malaysia. Feedback from the FMS who took part suggests that the classic pitfalls described in the literature were diminished, whilst benefits such as furthering personal development were achieved. On a wider scale, the authors believe that the implications for this study are that it could be applied to similar contextualisation processes for both undergraduate and postgraduate medical education, where the aim is to retain the advantages of an established home curriculum while ensuring it is also fit for purpose in the host country. Future study in this area is needed and may include revisiting this curriculum contextualisation in the future to assess its suitability over time and any further adaptations that have occurred or need to occur.

### Acknowledgements

The authors wish to acknowledge the participation of all the FMS who took part. Without their dedicated efforts applying their expert knowledge of primary care in Malaysia, this process would not have been possible. This study was funded through the MInTFM programme. MInTFM was involved in the design of the Delphi process for this study but played no further part in collection, analysis, and interpretation of data or in writing the manuscript.

### Conflict of Interest

The authors declare that they have no conflict of interest.

## Supplementary material

Supplementary file 1 Appendix. Questions asked of FMS around their experience of the process via an online survey tool
