# Supplementary material for: Cross-border contextualisation of family medicine curriculum: an examination of the process
Source: Int J Med Educ. 2023 Aug 31;14:117–22. doi: 10.5116/ijme.64e3.740e (PMC10693960; doi:10.5116/ijme.64e3.740e)
Supplement: Supplementary file 1 — Appendix. Questions asked of FMS around their experience of the process via an online survey tool [file ijme-14-117-S1.pdf]

Appendix

Questions asked of FMS around their experience of the process via an online survey tool

1. How did you find the process in contextualising the ICGP curriculum for Malaysian Family Medicine? Please expand to describe both negative and positive feelings and difficulties with the process.
2. What degree of consensus between the curriculum for Family Medicine in Ireland and Malaysia did you experience? Please expand.
3. In the areas of consensus between the curriculum for Family Medicine in Ireland and Malaysia what were the reasons for this consensus in your opinion?

In the areas of difference between the curriculum for Family Medicine in Ireland and Malaysia what were the reasons for this difference in your opinion?
